# Filamentous Phages As a Model System in Soft Matter Physics

**DOI:** 10.3389/fmicb.2016.01013

**Published:** 2016-06-30

**Authors:** Zvonimir Dogic

**Affiliations:** Department of Physics, Brandeis UniversityWaltham, MA, USA

**Keywords:** filamentous bacteriophages, soft matter physics, self-assembly, liquid crystals

## Abstract

Filamentous phages have unique physical properties, such as uniform particle lengths, that are not found in other model systems of rod-like colloidal particles. Consequently, suspensions of such phages provided powerful model systems that have advanced our understanding of soft matter physics in general and liquid crystals in particular. We described some of these advances. In particular we briefly summarize how suspensions of filamentous phages have provided valuable insight into the field of colloidal liquid crystals. We also describe recent experiments on filamentous phages that have elucidated a robust pathway for assembly of 2D membrane-like materials. Finally, we outline unique structural properties of filamentous phages that have so far remained largely unexplored yet have the potential to further advance soft matter physics and material science.

## Introduction

Nature is capable of assembling structures or remarkable complexity that frequently cannot be matched by materials synthesized in a laboratory setting. Filamentous phages are one example of such a material, where the knowledge of the fundamental biology has significantly advanced the seemingly unrelated fields of protein engineering and discovery, material science, nanotechnology and soft matter physics (Rakonjac et al., 2011). We review how filamentous phages have furthered our understanding of soft materials. Nature has engineered filamentous phages so that they are essentially identical to each other. Consequently properties of phage suspensions such as their length distribution are much narrower when compared to synthetic rodlike particles. This unique property has significantly impacted the field of soft matter physics directly leading to discovery of myriad structures, self-assembly pathways and previously unknown states of matter (Dogic and Fraden, 2006). We argue that the synergies and opportunities that emerge when filamentous phages are combined with soft matter physics have only been scratched and many exciting prospects lie ahead. The primary focus of this mini-review is on how filamentous phages have furthered our fundamental understanding of soft matter physics. Filamentous phages have also been used as a platform for developing materials with diverse practical applications ranging from biological sensors, piezoelectric and photovoltaic devices and phage-based batteries. These technologically important advances have been reviewed elsewhere (Mao et al., 2009; Rakonjac et al., 2011; Farr et al., 2014).

## Liquid crystalline phases of filamentous phages

**Figure 1:**
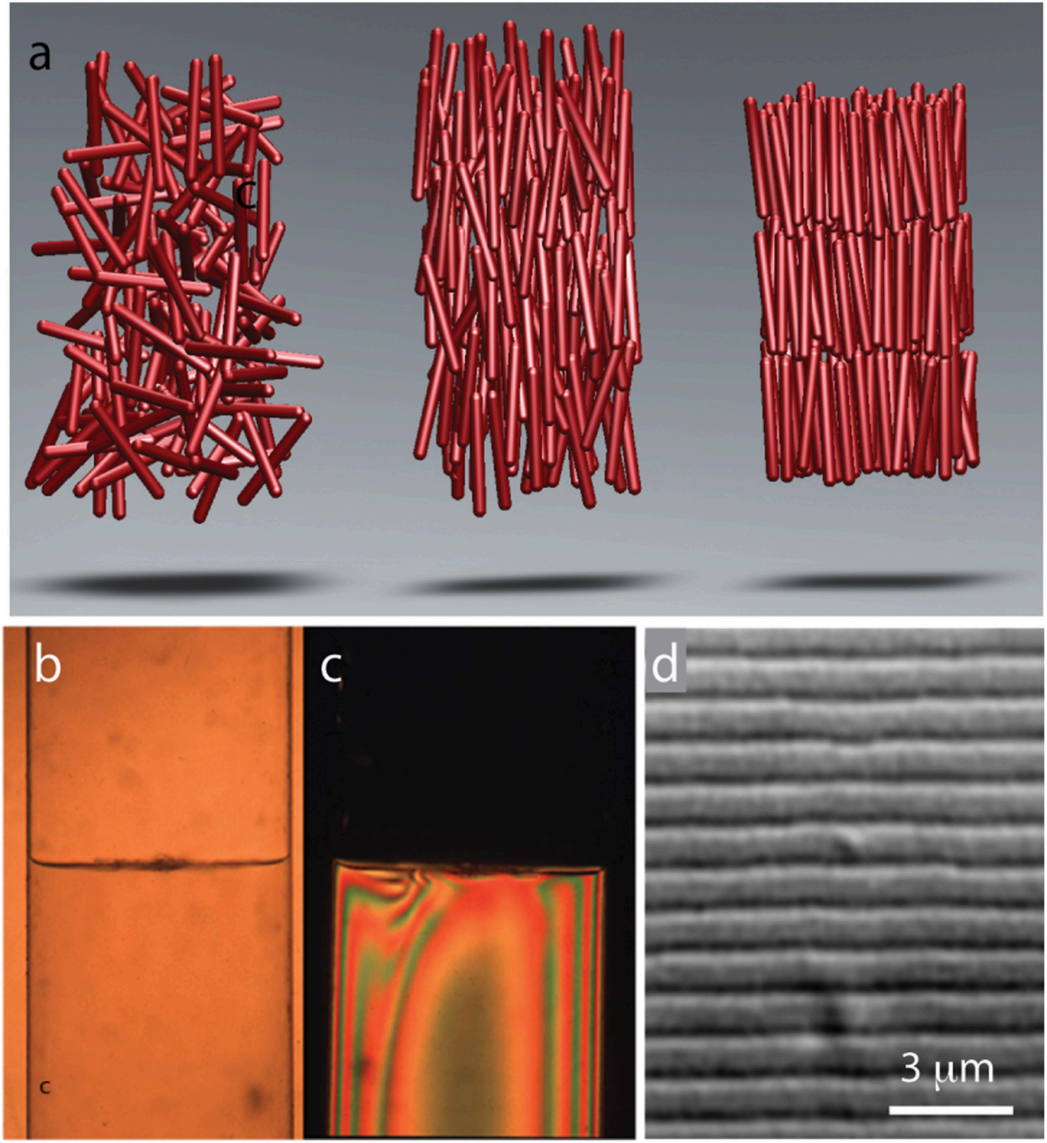
**Common liquid crystalline phases observed in colloidal suspension of rodlike viruses. (A)** Schematic illustration of rods in an isotropic, nematic, and smectic liquid phase. **(B)** With increasing concentrations a suspension of TMV particles exhibits coexistence between an isotropic and a nematic phase. **(C)** Image of the same coexisting isotropic-nematic sample viewed under cross-polarizers. The birefringent nematic phase with higher density sediments to the bottom. **(D)** At high concentrations filamentous viruses form a smectic phase. High-resolution differential interference contrast (DIC) microscopy allows for direct visualization of one-rod-length long smectic layers. Here we illustrated the most common and important liquid crystalline phases. Filamentous phages exhibit a much richer phase diagram (for more details see Lettinga et al., 2005; Lettinga and Grelet, 2007; Pouget et al., 2011; Naderi et al., 2013).

Gels as well as suspensions of colloids, polymers, and amphiphiles are typical examples of soft materials. Liquid crystals, comprised from anisotropic rod-shaped molecules, are another technologically important example (de Gennes and Prost, 1995). At low concentrations, rod-like molecules are disordered and point in random directions. Such an arrangement of rod-like molecules is called an isotropic phase (Figure 1A). With increasing particle concentration an isotropic phase undergoes a discontinuous transition into a phase, in which all rods are aligned along one particular axis. Such an arrangement of molecules is called a nematic liquid crystal. Increasing the particle concentration further leads to more ordered phases. Of particular prominence is a smectic phase in which the molecules organize into one-rod-length thick liquid-like layers of aligned rods that are stacked on top of each other (Figure 1A). In the language of condensed matter physics smectic liquid crystals have a long-range orientational order and 1D quasi-long-range positional order.

In many ways filamentous viruses have catalyzed the development of the entire field of colloidal liquid crystals. In 1935, Wendell Meredith Stanley purified anisotropic rod-like Tobacco mosaic virus (TMV) (Stanley, 1935). Soon thereafter, John Bernal and his coworkers prepared TMV suspensions at high-enough concentrations to observe formation of a stable nematic phase (Bawden et al., 1936). Amongst others, these observations have inspired Lars Onsager to develop his seminal theory of the isotropic-nematic phase transition in colloidal liquid crystals (Onsager, 1949). However, TMV has a number of deficiencies including: long time required to grow tobacco plants and isolate the virus, the relative ease with which particles aggregate end-to-end yielding polydisperse suspensions, and the difficulty of introducing genetic modifications. For these reasons filamentous phages have supplanted TMV as a choice model system of rod-like particles.

While liquid crystalline behavior of filamentous bacteriophages has been known to structural biologists for a long time, the first detailed report describing these properties has been published by the group of Don Marvin (Lapointe and Marvin, 1973). Amongst other information this work reports direct visualization of smectic layers, a feat that precedes by many years the imaging revolution that has profoundly affected soft matter physics. Since these early advances the filamentous phages have become an important model system used to investigate liquid crystals. For example, filamentous bacteriophages enabled the first quantitative test of the Onsager theory. Specifically, once the virus flexibility and surface charge is taken into account, it was shown that the Onsager theory quantitatively explains the location and nature of the isotropic to nematic phase transition (Figures 1B,C; Tang and Fraden, 1995; Barry et al., 2009). Large aspect ratio and length uniformity were the two essential features that enabled quantitative comparison between experiments and theory.

While many biological and synthetic systems exhibit nematic ordering, observing a smectic phase has been a more formidable challenge. Since smectics are layered structures it is essential that the constituent rodlike molecules have uniform length. Until very recently, these conditions have been difficult to realize in synthetic systems (Kuijk et al., 2011). Therefore, smectic ordering in colloidal liquid crystals was first observed in TMV suspensions in the 1980's (Wen et al., 1989). Following upon this early work, phases with smectic-A, crystalline and columnar order have been more thoroughly characterized in suspensions of filamentous bacteriophages (Figure 1D; Dogic and Fraden, 1997; Grelet, 2008, 2014). Comparison between the rigid TMV rods, semi-flexible *fd* rods, theoretical models and computer simulations has demonstrated that even a slight flexibility pushes the nematic-to-smectic phase transition to higher particle concentrations and suppresses the formation of a smectic phase. Isolated filamentous phages can be visualized with optical microscopy, an advance that has provided important insight into the dynamics of the nematic, smectic, and columnar liquid crystals (Lettinga et al., 2005; Lettinga and Grelet, 2007; Pouget et al., 2011; Naderi et al., 2013).

## A more recent example—assembly of phage based 2D colloidal membranes

So far we have described how the unique properties of filamentous bacteriophage suspensions have been used to test long-standing theoretical predictions related to bulk ordering of rod-like particles and thus advance the field of colloidal liquid crystals. More recent experiments, which again have relied on the unique properties of virus particles, have elucidated a fundamentally different pathway for assembly of virus particles into 1D and 2D materials. We discuss some of these advances and how they impacted our broader thinking about self-assembly of soft materials.

**Figure 2:**
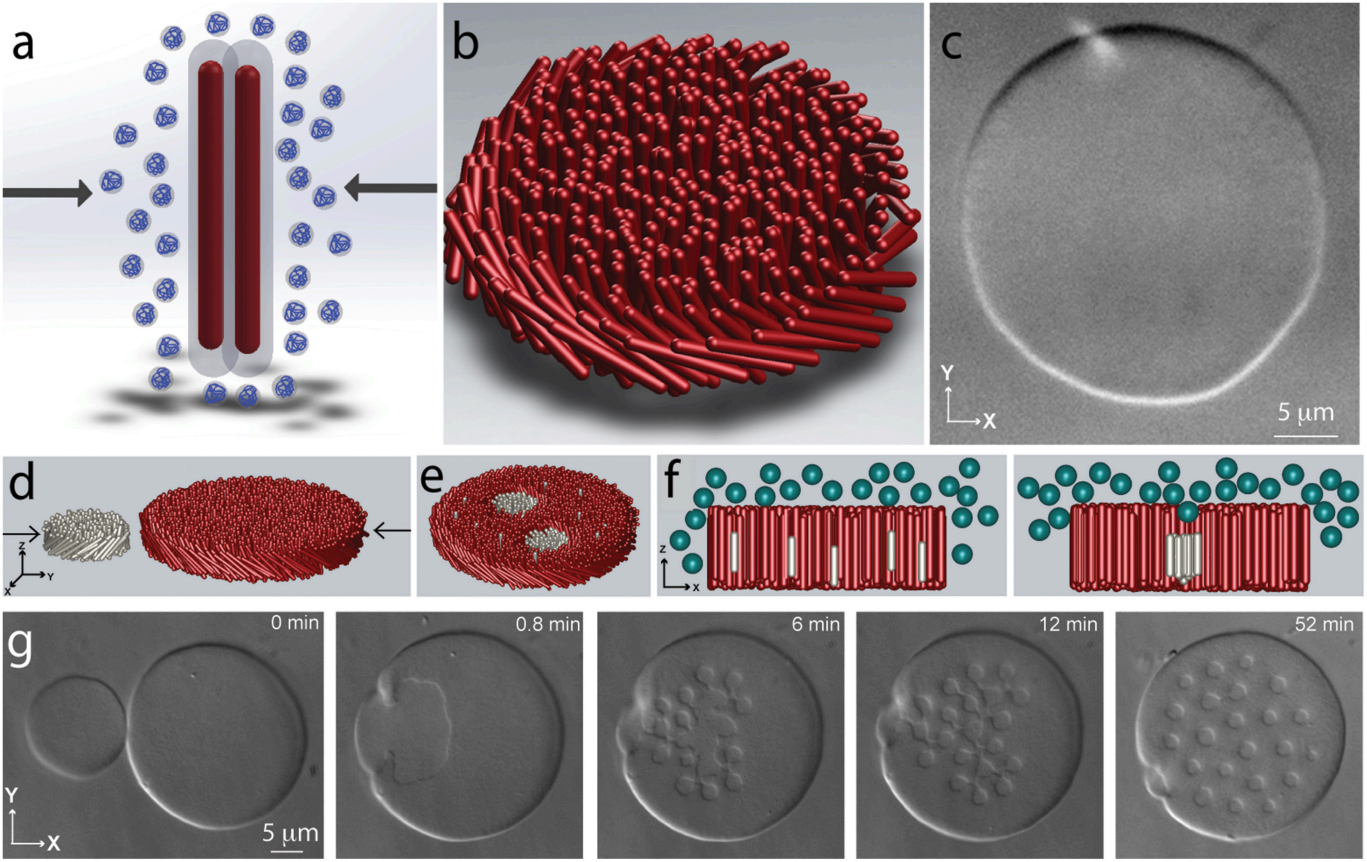
**Assembly of filamentous phages into 2D colloidal membranes and rafts. (A)** Schematic illustration of the depletion interaction. Addition of non-adsorbing polymer leads to the imbalance of the osmotic pressure and effective attractive interactions between rod-like particles. **(B)** Depletion interactions lead to lateral condensation of rod-like particles and formation of 2D colloidal membranes that are enveloped by the depleting polymer suspension (not shown in the schematic). **(C)** DIC microscopy image of a colloidal membrane comprised from *fd-wt*. Rod-like particles point along the *z*-direction and the assemblage is comprised of millions of rod-like viruses. **(D)** Coalescence of two colloidal membranes leads to robust assembly of finite-sized colloidal rafts. Each membrane is comprised of filamentous phages of different length and chirality. Each monodisperse raft contains approximately 20,000 virus particles. **(E)** Schematic illustration of the coalescence experiments. **(F)** Lateral association of shorter rods dissolved within a colloidal membrane can be understood in terms of excluded volume. Inserting an isolated rod into a membrane creates additional volume at the ends of the filament that is inaccessible to the depleting polymer. Lateral association of shorter rods reduces the excluded volume and thus decreases the system free energy. **(G)** Lateral coalescence of short right-handed fd-Y21M and long left-handed M13KO7 membranes leads to the formation of finite-sized rafts.

Virus particles are traditionally purified through a few cycles of differential centrifugation. In a first step a non-adsorbing polymer such as polyethylene glycol (PEG) is added to a dilute virus suspension. This induces filament attraction and their condensation, causing the suspension to acquire a cloudy appearance. This ubiquitous effect is know as depletion attraction in colloid science and macromolecular crowding in biology (Figure 2A; Asakura and Oosawa, 1954; Zhou et al., 2008). Recent work has shown that in purified virus suspensions the same effect leads to formation of myriad assemblages with surprisingly complex shapes (Dogic and Fraden, 2001; Dogic, 2003). At lowest depletant concentrations the viruses condense into spindle-shaped droplets (Dogic, 2003). Within the droplet the rod-like particles have nematic structure. Increasing the depletant concentration leads to a dramatic shape change of the condensed droplet. In particular, at intermediate depletant concentrations the viruses associate laterally into a one rod-length thick monolayer membrane comprised from aligned rods (Figures 2B,C). The lateral size of these quasi-2D assemblages can be hundreds of microns (Barry and Dogic, 2010; Gibaud et al., 2012; Yang et al., 2012; Zakhary et al., 2014). Within each layer rods retain liquid-like dynamics. The properties of such 2D-assemblages are similar to those of conventional lipid bilayers, hence such structures are called colloidal membranes. Finally, at highest depletant concentrations the 2D colloidal membrane layers stack on top of each other, thus forming a bulk 3D condensate with smectic-like structure.

The discovery of colloidal membranes is important for several reasons. From a general perspective, it is possible to think of colloidal particles as giant atoms, and there is a tradition of exploiting an analogy between colloidal and molecular systems. For example, with increasing concentration micron-sized spherical colloids spontaneously transition into 3D crystal (Pusey and Vanmegen, 1986). Many properties of such colloidal crystals bear resemblance to more conventional crystals built from molecular building blocks. However, in contrast to molecular crystals, optical microscopy enables visualization and tracking of all the constituent colloidal units as the crystal undergoes complex dynamical processes. Consequently, colloidal crystals have provided insight into universal processes in crystals that do not depend on the size of the constituent units, such as nucleation dynamics and glass formation (Gasser et al., 2001; Schall et al., 2004; Alsayed et al., 2005).

In a similar vein, although the microscopic interactions that drive their assembly are distinct, colloidal monolayers are micron-sized analogs of conventional lipid bilayers. Most importantly the same class of theories describe large-scale continuum deformations of both colloidal monolayers and lipid bilayers (Safran, 1994; Barry and Dogic, 2010). It follows that the studies of colloidal membranes could provide important insights into the universal aspects of membrane biophysics. For example, it has been suggested that lipid rafts, which are nanometer-sized clusters of certain lipids and other membrane soluble proteins, are the fundamental organizing principle of biological membranes (Lingwood and Simons, 2010). However, because of the size of the elemental units it is not possible to visualize lipid rafts and the subject remains controversial. From a more general perspective little is known about how membranes mediate effective interactions between various inclusions. We note that the important features of constituent viruses, namely their large and highly uniform length, endow colloidal membranes with their unique properties. Most importantly, colloidal membranes are easily visualized by optical microscopy offering a new system that might provide insight into the nature of ubiquitous membrane mediated interactions.

Recent experiments have explored the behavior of inclusions embedded within a colloidal monolayer membrane (Sharma et al., 2014). Specifically, colloidal membranes were doped with a low volume fraction of rods that were 30% shorter than the background rods. At low depletant concentrations the shorter rods where uniformly dispersed throughout the colloidal membrane, while at high osmotic pressure they bulk phase separated from the background membrane. Intriguingly, at intermediate depletant concentrations the shorter rods associated into robust, permanently stable, finite-sized clusters, comprised from approximately 20,000 rods (Figures 2D–G). The unique lengthscale of colloidal membranes have enabled characterization of colloidal rafts in great detail. For example, a combination of single-molecule tracking and FRAP techniques enabled direct measurement of *k*_*on*_ and *k*_*off*_, the kinetic rates at which individual rods associated with and dissociate from a colloidal raft. From here it is possible to measure the free energy landscape of colloidal rafts. Similar measurements are not feasible in analogous molecular systems. Using optical tweezers it was also possible to directly trap colloidal rafts and measure their membrane-mediated interactions. These experiments have shown that colloidal rafts have long-ranged repulsive interactions that can be many microns. Furthermore, it has been possible to relate these effective interactions to the distortions of the background membrane that can be visualized using specialized optical microscopy techniques. Again these experiments were only possible due to the unique properties of colloidal membranes.

It is intriguing to speculate about the possible broader relevance of such findings. For example, colloidal rafts demonstrate that all chiral inclusion in a colloidal membrane will acquire long-ranged repulsive interactions. Since these interactions depend only on the symmetries of the constituent particles, membrane-mediated chiral repulsions are universal and relevant for both lipid and colloidal membranes. Intriguingly, for conventional membranes, it is known that cholesterol is essential for assembly of lipid rafts. A possible and largely unexplored role of cholesterol might be to induce local twist, which would lead to raft-raft repulsions, and thus stabilize finite size assemblages. Intriguingly, from liquid crystal literature cholesterol is known to be an effective chiral dopant; hence chiral nematics are referred to as a cholesteric phase (de Gennes and Prost, 1995).

Self-assembly of membranes is a ubiquitous process in nature whose importance spans disparate scientific fields and many technological applications. The paramount example of lipid bilayers has inspired the synthesis of numerous other building blocks that assemble into 2D membrane-like structures (Discher et al., 1999; Antonietti and Forster, 2003; Park et al., 2004). The common feature shared by all these systems is the covalent link that holds together chemically incompatible moieties, such as a hydrophobic chain and a hydrophilic head. This irreversible bond introduces a molecular frustration that suppresses macroscopic bulk phase separation, and instead drives the formation of microphase-separated structures (Israelachvili, 2011). From this perspective, colloidal membranes are important for several reasons. First, they establish a new route for solution based self-assembly of 2D materials, one that does not rely on the established paradigm of amphiphilic assembly of chemically heterogeneous moieties, but rather on the geometrical properties of the constituent particles. Second, the formation of colloidal membranes is driven by universal hard-core repulsive interactions. Since such exclude volume interactions are relevant to all rod-like particles, experimental findings using filamentous bacteriophages should be widely applicable.

## Opportunities and challenges that lie ahead

The uniformity of particle length has made filamentous bacteriophages an attractive model system in soft matter physics. However, there are other appealing features of filamentous phages that could also impact soft matter physics, but have not been utilized to nearly the same extent. Further advances will likely require a much tighter cooperation between biologist that can systematically modify various virus properties and physicist who can use these particles to assemble and characterize novel soft materials.

Filamentous phages afford almost arbitrary control over their contour length, which is another exciting feature. Because its length scales linearly with its genome size, filamentous phages have a unique ability to package very long or short DNA segments. Longer genomic DNA lead to longer viruses. Using molecular cloning techniques it has been possible to engineer viruses that are as short as 50 nm and as long as 8000 nm (Herrmann et al., 1980; Specthrie et al., 1992; Marchi et al., 2014; Brown et al., 2015; Sattarl et al., 2015). In principle, this makes it feasible to create monodisperse rods whose aspect ratio varies from anywhere between 10 and 1000. So far the filamentous phages with tunable lengths have been used in only a few studies, starting with the pioneering experiments that examined how rotational dynamics of rods in the isotropic phase scales with their length (Maguire et al., 1980). Subsequently, viruses have been used to examine the effect of rod length on the location of the isotropic-nematic phase transition as well as the stability of the smectic phase (Dogic and Fraden, 2001; Purdy and Fraden, 2004, 2007). Furthermore, studies on colloidal membranes have demonstrated dramatic effect that even slight changes in the rod lengths have on the stability of colloidal clusters (Sharma et al., 2014).

However, these studies have only scratched the surface of many experiments that become possible with a library of viruses of different lengths. Creating such a library requires expertise in molecular cloning that is not readily accessible to the physics community. A particular challenge is that longer phages have a tendency to delete the inserted DNA segments and thus revert back to the wild-type length. Since longer phages take longer to grow any particle that excises the extra DNA segment has shorter reproduction time and quickly overtakes the entire population. However, there are very few alternate model systems of rod-like particles with tunable length, and creating a library of filamentous phages of varying contour length that can be robustly grown would surely have a significant impact on soft matter physics.

Another attractive feature of the phages that has not received enough attention is the possibility of tuning the physical properties of the filaments by systematically changing the composition of the major coat protein. For example, structural biologists have studied fd-Y21M (a mutant virus in which the 21st amino acid of the major coat protein is changed from tyrosine to methionine) because it yielded higher quality diffraction patterns (Marvin et al., 1994). More recent work has demonstrated that Y21M mutation dramatically changes the physical properties of the virus (Barry et al., 2009). In particular, the persistence length of Y21M mutant is 9.9 μm in comparison to the wild-type phage that is only 2.4 μm. Furthermore, *fd-wt* forms a cholesteric liquid crystal with left-handed chirality, whereas Y21M mutant switches the handedness of the system. Needless to say the phase space of all possible mutations of the major coat protein is much larger, and so far there has been no attempts to systematically alter the coat protein structure and correlate this to coarse-grained properties of the filament. For example, it would be interesting to determine how the bending rigidity of filaments changes as the composition of the major coat protein varies from purely Y21M to wild-type protein. This might systematically change the bending rigidity and thus create filaments with tunable persistence length and chirality, a unique feature not present in any other model system. Using phage display technology it has proven possible to systematically change the composition of the coat proteins within a single virus (Rowitch et al., 1988).

Finally, another underused but attractive feature of filamentous bacteriophages is the structure of its ends. Specifically, a distinct set of proteins is associated with each virus end, thus raising the possibility of specifically labeling each virus end. There have been several notable efforts in this direction, although most of these have been restricted to studies at a single filament level instead of studying properties of macroscopic assemblages. In an important early step, phage display technology has been used to select for amino-acid sequences that specifically bind to inorganic surfaces (Whaley et al., 2000). This feature made it possible to assemble soft materials in which filamentous phages organize into smectic layers that are intercalated with layers of end-bound inorganic nanoparticles (Lee et al., 2002). It should be noted that there are alternate pathways toward assembly of such structures. In particular, similar lamellar structures can also robustly form in a simple mixture of hard rods and spheres that lack any specific link between spheres and rods (Adams et al., 1998; Dogic et al., 2000). In another notable study two ends of the virus have been labeled with distinct labels. In one case these molecules associated with each other, thus transforming linear bacteriophages into ring-like structures, while in another experiment similar end labels have been used for single-molecule experiments that have measured the mechanical properties of the virus using force-extension experiments (Nam et al., 2004; Khalil et al., 2007). Finally, in another study the end labeled viruses have been used to coat the surface of a micron-sized colloidal particle and thus create a unique star colloids with very long repulsive interactions (Huang et al., 2009). However, these are only a few of many applications that become possible using end labeled filaments. In particular, a chemical coupling scheme is needed that will allow for specific and robust attachment of diverse sets of chemicals, including dyes to either of the filaments ends. Such a scheme would enable a much wider range of experiments.

## Author contributions

The author confirms being the sole contributor of this work and approved it for publication.

## Funding

Research program on self-assembly of filamentous viruses is supported by the National Science Foundation grants no. DMR-MRSEC-1402382, DMR-1609742 and DMR-0955776.

### Conflict of interest statement

The author declares that the research was conducted in the absence of any commercial or financial relationships that could be construed as a potential conflict of interest.
